# Quantification of Dry Matter Content in Hass Avocado by Near-Infrared Spectroscopy (NIRS) Scanning Different Fruit Zones

**DOI:** 10.3390/plants12173135

**Published:** 2023-08-31

**Authors:** Pablo Rodríguez, Jairo Villamizar, Luis Londoño, Thierry Tran, Fabrice Davrieux

**Affiliations:** 1 Research Unit ITAV: Innovaciones Tecnológicas para Agregar Valor a Recursos Agrícolas, Sector Llanogrande, Centro de Investigación La Selva, Corporación Colombiana de Investigación Agropecuaria-Agrosavia, km. 7, Vía Rionegro—Las Palmas, Rionegro-Antioquia 054048, Colombia; javillamizar@agrosavia.co; 2 International Center for Tropical Agriculture (CIAT), Valle del Cauca, Palmira 763537, Colombia; l.londono@cgiar.org; 3 UMR Qualisud, Univ Montpellier, Avignon Université, Centre de Coopération Internationale en Recherche Agronomique pour le Développement (CIRAD), Institut Agro, Institut de Recherche pour le Développement (IRD), Université de La Réunion, F-34398 Montpellier, France; thierry.tran@cirad.fr (T.T.); fabrice.davrieux@cirad.fr (F.D.)

**Keywords:** dry matter, near-infrared spectroscopy, Hass avocado, fruit composition gradient, fruit quality, *Persea americana*

## Abstract

Accurate dry matter determination (DM) in Hass avocados is vital for optimal harvesting and ensuring fruit quality. Predictive models based on NIRS need to capture fruit DM gradient. This work aimed to determine the DM content in Hass avocado whole by NIRS scanning different fruit zones. Spectra were recorded for each zone of the fruit: peduncle (P), equator (E), and base (B). The calibration and validation included fruit from different orchards in two harvest cycles. The results show a DM gradient within the fruit: 24.47% (E), 24.68% (B), and 24.79% (P). The DM gradient was observed within the spectra using the RMSi (root mean square) criterion and PCA. The results show that at least one spectrum per fruit zone was needed to represent the variability within the fruit. The performances of the calibration using the whole set of data were R^2^: 0.74 and standard error of cross-validation (SECV) = 1.18%. In the validation stage using independent validation sets, the models showed similar performance (R^2^: 0.75, SECV 1.15%) with low values of the standard error of prediction (SEP): 1.62%. These results demonstrate the potential of near-infrared spectroscopy for high-throughput sorting of avocados based on their commercial quality.

## 1. Introduction

Harvesting Hass avocados is challenging due to their complex physiology and accumulation of solid material during fruit development. Many studies note that it is necessary to have a methodology or adopt technologies that ensure that avocado quality is consistent across all stages of the supply chain [1]. Researchers have reported that preharvest factors influence the fruit composition of Hass avocados [2]. The dry matter (DM) of fruit pulp is the most widely used indicator internationally to determine quality attributes and minimize defects in fruit pulp [3]. There are other harvest maturity indicators that have been used to evaluate quality attributes, with the oil content (OC) being the most studied to identify the ideal harvest time. However, Lee et al. noted that due to low costs and rapid determination, DM can be considered the standard indicator for harvest maturity due to its close relationship with fruit OC [4].

Research has shown that a minimum DM of 24% is a reliable indicator of harvest maturity for Hass avocados in different countries [4,5,6]. After this value, the sensory quality improves, and the internal disorders are reduced in the range of 26–28% of DM. In the late harvest season (DM > 28), there is an increase in internal fruit damage and a high probability of losses due to rot [7]. However, in the same orchard, there may be several flowering cycles, leading to fruit with different dry matter contents at the time of harvest, and the harvester is not able to differentiate these differences with the naked eye. Moreover, most laboratory-based techniques destroy the fruit samples in order to classify them, are time-consuming, and fail to represent DM variability in orchards; thus, nondestructive alternatives, such as near-infrared spectroscopy (NIRS), can be useful for quickly predicting maturity parameters [8].

NIRS has been used to evaluate the quality of fresh agricultural produce, including fruit maturity analysis [9]. NIRS can be used in both reflectance and interactance modes to establish the DM content in Hass avocado fruit [10,11]. NIRS works by measuring the difference in intensity between transmitted and received light delivered at specific wavelengths. In fruit maturity analysis, NIRS can be used to evaluate the firmness of peaches [12]. The use of NIRS has also been proven to be effective in predicting the total acidity of intact mango. NIRS can also be used to evaluate the quality of avocado fruit, including parameters such as oil content and moisture content [13]. NIRS is a promising technique for analyzing avocado composition because it provides information about C-H, O-H, and N-H bonds. Avocado quality parameters, like dry matter (DM), moisture, and oil content, depend on organic molecules containing C-H, O-H, C-O, and C-C bonds. Therefore, NIR technology holds the potential for accurately quantifying these parameters in avocados [14]. Chemometric analysis is integral to NIRS technology, involving multivariate analysis for interpreting extensive datasets of NIR spectra. Initially, a calibration model is developed by correlating spectra with conventional destructive data. This entails employing diverse chemometric tools, such as partial least square regression (PLS), multivariate linear regression (MLR), and principal component regression (PCR). Among these, PLS is the preferred choice due to its capability to exclude latent variables (LVs) inconsequential for explaining variance in targeted quality attributes [8].

When using measurement equipment where the response variable is reflectance, the change in harmonic vibrations that occur in the region (Vis-NIRS) is represented and stored as a record of reflectance (1/R) versus wavelength [15]. Most studies perform NIRS measurements directly on the skin (exocarp), as mentioned by Walsh et al. in their studies, in which they highlight that the scans are performed in diffuse reflection or interaction mode to prevent any damage; this method is considered a nondestructive technique, allowing NIRS radiation to penetrate inside and capture all the properties of the fruit pulp [16].

Several NIRS studies have been conducted to evaluate the implementation of nondestructive techniques that identify harvest maturity indices in Hass avocados. However, due to the DM gradient present in the fruits, representing this variability is essential for obtaining reliable results. The first studies carried out by Schroeder showed that there is a pronounced DM gradient that is dispersed throughout the fruit [17]. This internal variability can be associated with development and maturation problems for the fruit on the tree. It has also been reported that there is a gradual decrease in DM from the end of the peduncle toward the interior of the fruit near the seed and from the sides to the interior of the fruit [18].

However, the different studies developed to predict DM by NIRS do not establish a clear methodology that allows recovery of all the variability present in the fruit. Wedding et al. implemented two scans per fruit over the range of the peduncle to the basal zone (equator), finding that the performance of the predictive model for DM only stabilizes if several seasons or harvests are considered to collect a greater variability in fruits [19]. Other researchers carried out between four and six equidistant scans around the equator zone of each fruit to evaluate nondestructive models and predict the DM in Hass avocado fruits [20,21], and their methodology was unlike the methodology implemented by Olarewaju et al., who implemented two scans in the equator zone after rotating the fruit 180° and averaged the spectra to perform the prediction models [8]. Moreover, in Mexico, nondestructive studies were carried out with scans on the peduncle to obtain more reliable results than those evaluated in the equatorial zone due to the interference of the DM gradient present in the seed [22]. Our approach acknowledges the inherent variability found in Hass avocado fruit due to its overall composition, which significantly impacts its internal quality and ripening patterns. Most of the physiological and pathological changes that affect fruit quality occur in the peduncle.

Since consumers evaluate fruit quality as a whole and consume the fruit in its entirety, it is essential to include all zones (peduncle, equator, and base) of the fruit to accurately determine its dry matter content, either by the reference method or nondestructive techniques. This study aimed to employ destructive analyses and NIRS to determine dry matter content at various fruit zones—peduncle, equator, and base—enabling the creation of predictive models for accurately assessing overall dry matter content in Hass avocados.

## 2. Results and Discussion

### 2.1. Number of NIRS Scans Per Fruit Zone for Assessing the Dry Matter Gradient in the Whole Fruit

**Figure 1:**
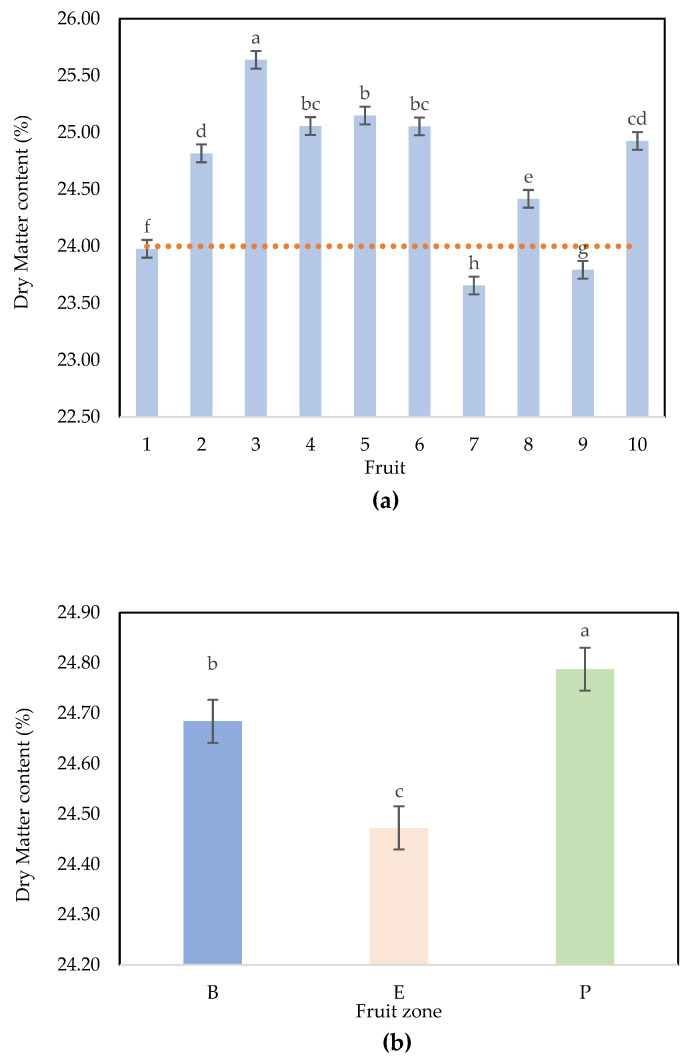
Dry matter variation in the samples (**a**) and fruit zones (**b**). Dotted orange line: minimum DM needed to harvest Hass avocado to guarantee internal quality [6]. Different letters represent significant differences (*p* < 0.05) using ANOVA and Tukey post hoc. Fruit zone: B: base, E: equator, P: peduncle.

The DM values range between 23.41% and 25.73%, with an average value of 24.65% and a standard deviation of 0.65%. The repartition of the values shows two populations and is in concordance with the discussion that is presented below (Figure S1). This repartition is due to fruits F1, F7, and F9, which present average DM contents lower than 24% (see Figure 1a).

The ANOVA showed that the probability of Fisher’s F test was significant (*p* < 0.0001); therefore, there was a significant effect of the factors (zone and fruit) on the DM content (Table 1). According to the sum of squares and Fisher’s exact test, the factor “fruit” effect was more significant than a “zone” effect based on type III errors. Thus, to effectively represent the DM variability inside Hass avocado samples, the fruit zone orientations in the analysis needed to be considered to determine the maturity at different stages in the value chain (before harvesting, at harvest, and once the fruit arrives in the packing house, etc.).

The Newman–Keuls (SNK) post hoc test comparing the differences between the factor “fruit” with a confidence interval of 95% confirms that fruits 3, 8, 1, 9, and 7 are different from all other fruits and that fruits 4, 5, and 6 are not significantly different (same DM); the same result occurs for fruits 4, 6, and 10 and for 10 and 2 (Figure 1a). A Newman–Keuls (SNK) test comparing the differences between the levels of “zone” with a confidence interval of 95% confirms that the three zones are significantly different, with average values of 24.47% (equator), 24.68% (base) and 24.79% (peduncle) (Figure 1b).

There is a DM gradient within the fruit, with higher DM content on the peduncle followed by the equator and base zones. The variability within the fruit was shown by Phetsomphou and Wedding et al. [11,23] not only in the P, E, and B zones but also in the outer, middle, or inner fruit. Therefore, this gradient affects the robustness of both destructive and nondestructive analyses for DM quantification. The literature does not show a clear trend in the direction of the gradient. Moreover, there is a lack of information that explains the phenomenon well. Schroeder indicated that these gradients could be related to complex fruit development physiology [17].

There are many differences in structure and metabolic activity that eventually are demonstrated within the avocado fruit. Moreover, the gradient within the fruit and the variability between fruits increase the difficulty of guaranteeing the minimum dry matter needed to export fruits. According to Rodríguez et al., almost 80% of fruit samples must have a DM higher than 24% to have good postharvest quality in the international market [6]. The results show that 70% of samples in this study have DM higher than 24%.

Regarding methodologies applied to build NIRS predictive models of Hass avocado DM estimation, almost all the authors scanned the fruit in the equatorial zone two or more times [8,10,19,24]. In other fruits, the same methodology has been employed in that only the equator zone is scanned [25,26]. Although the DM gradient within the fruit has been published for Hass avocados, NIRS models are considered destructive analyses of the outer (0.5–1.0 cm) layer of the mesocarp and skin; thus, the analysis could have a bias with respect to the whole fruit DM content. Subedi and Walsh found a difference of 4% in DM between the outer and inner parts of Hass avocados [24]. The authors developed predictions despite this high variation. In terms of fruit development, 4% dry matter implies a difference in fruit age of almost 40 days, according to Rodríguez et al. [6]. We found a nonsystematic bias in the dry matter of ±4% in the analysis of the DM with commercial, portable NIRS in Colombia. Therefore, these devices do not allow efficient sorting of fruit in orchards or packing houses.

**Figure 2:**
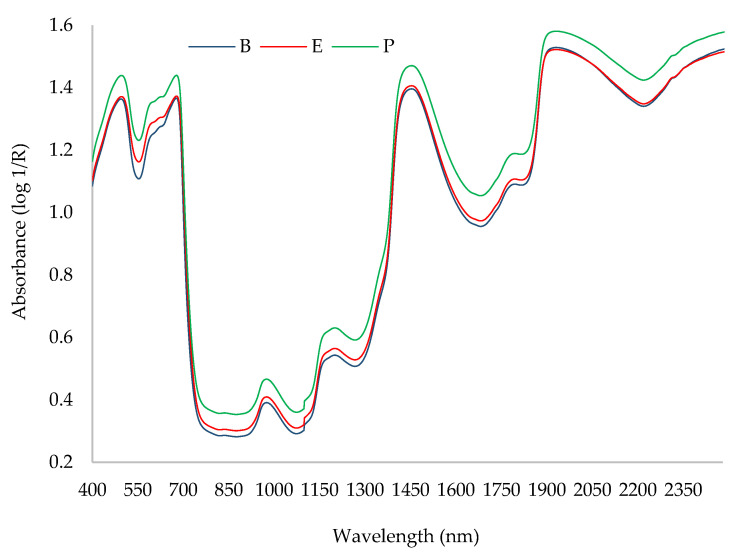
Average spectra obtained from each zone of the Hass avocado fruit. Fruit zone: B: base, E: equator, P: peduncle.

Figure 2 shows an average representative absorption spectrum for the Hass avocado fruit before spectral preprocessing, categorized by fruit zone. The absorption bands in Figure 2 corresponded to specific wavelength regions. The first bands were observed in the visible region (420–750 nm) and associated with the green (500–600 nm) and red (625–750 nm) spectral ranges [27]. The second absorption band was observed between 930 and 1030 nm and associated with the O-H bond commonly found at 960 nm in spectra from fresh plant tissues, indicating a high moisture content of the fresh avocado fruit [28,29]. Based on other studies [8,10,30], the spectral regions that corresponded to the stretching and combinations of C-H_2_ related to oil were identified as the wavelength bands located between 900 and 920 nm, at approximately 930 nm, 1200 nm, and 1750 nm, and between 2200 and 2400 nm. Our average spectra were similar to other published results [8,31]. However, this is the first study to show spectral differences between the fruit zones within the Hass avocado fruit via the NIRS technique.

**Figure 3:**
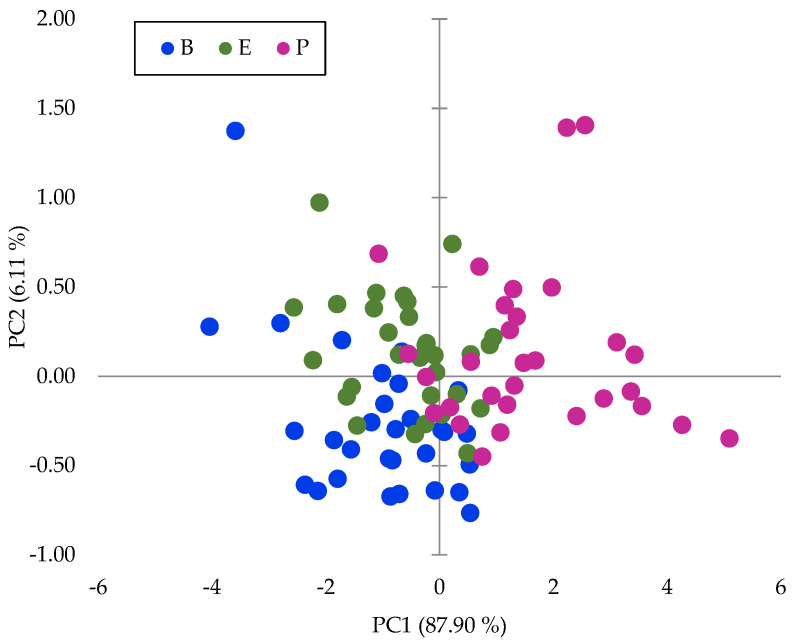
Sample scores for the first two PCs in the principal component analysis of the NIRS raw data. Fruit zone: B: base, E: equator, P: peduncle.

PCA was performed on the raw data, log (1/R), for the whole wavelength range, and the dimension of the input matrix is n = 90 and *p* = 1050. The first two PCs explained 94% of the total inertia and 88% and 6%, respectively. The presentation of the sample scores for the first two PCs highlights differences between zones (Figure 3), which indicates that there is high variability within spectra due to the zone of the fruits. These groups have not been reported before, although there are many publications on the use of NIRS technology to analyze DM in Hass avocados.

**Figure 4:**
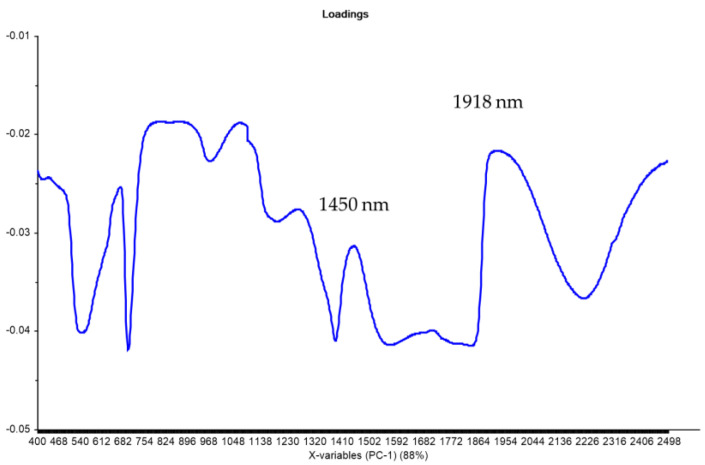
Loading plot associated with PC1 of the NIRS raw data.

The loadings associated with PC1 show two main peaks in the NIR region: 1450 nm and 1918 nm, which correspond to water (O-H) absorption bands (Figure 4). The results agree with those of other works that indicated that for Hass avocados, the main peaks are closely associated with the H-O-H stretching modes of water [8].

Among the most common set of data science tools applied in analytical NIR, PCA (principal component analysis), a dimensionality reduction method, should also be mentioned. It is commonly used in exploratory data analysis to reduce the complexity of high-dimensional datasets by transforming the original variables into a smaller set of new variables, called principal components, which explain most of the variation in the data [32]. It can be used to analyze the quality parameters of apples by spectroscopy from Vis/NIR to the NIR region. In the context of cultivar identification, PCA was able to retain 98% of the data for apple cultivation, making it an ideal feature extraction method [33]. Our findings confirm the utility of PCA analysis to find patterns in NIR spectroscopy datasets.

The RMSi for each zone (Figure S2) shows that despite some outlier spectra (with high RMS values), all RMSi values are of the same order.

The RMSi average values are given per zone (for all the fruit), and an ANOVA on the RMSi values with zone as factor confirms that there is a significant effect (α = 5%) of the zone of the fruit on spectra variability (Pr > F = 0.043), the contrast test (Newman–Keuls, SNK) confirms a difference between peduncle and equator, and no difference between peduncle and base and between base and equator occur (Figure S3). These results associated with DM content observations suggest that at least one spectrum per zone is reasonable for determining the variability in the whole fruit.

The RMSi method of calculating dispersion is commonly used in NIRS analysis and is known as the root mean square error or root mean square deviation. It is a measure of the variability in a group of spectra that are supposed to be similar. Therefore, this descriptor indicates spectral similarities between avocado zones. This study highlights the existing spectral variability between fruit zones. Therefore, at least one scan must be performed per zone to capture the whole fruit variability.

### 2.2. Application of Different NIRS Scans by Fruit in Maturity Monitoring of Hass Avocados

#### 2.2.1. Calibration

In the second experiment, the laboratory method yielded DM results ranging from 18.73% to 27.42% for both harvests (2022a and 2022b). This range includes values that have been identified as typical for commercial Hass avocado harvests in Colombia. Various pretreatments were tested to determine the optimal one based on lower SEC and SECV values, as well as higher R^2^. In the complete dataset, 18 outliers were identified, accounting for a low percentage of the total samples (2.5%). These outliers were subsequently removed from the PLS analysis.

**Figure 5:**
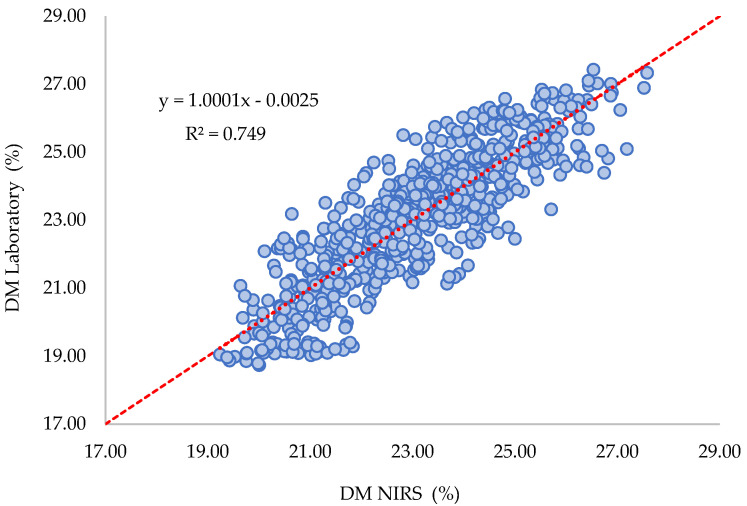
DM measured by the oven method versus DM predicted by the PLS model based on NIRS spectral fingerprints.

The analysis revealed that the best results, with an R^2^ of 0.75%, were obtained using 13 LVs (latent variables) (see Figure 5). The SEC was 1.0%, indicating adequate precision, as is discussed later. The estimated range for DM using NIR spectroscopy in cross-validation was found to be from 19.14% to 27.58%, which is satisfactory for practical applications within the commercial harvest range in Colombia. The SECV was 1.18%, indicating an acceptable level of accuracy, and the total number of outliers was 2.5% of the total number of samples, which is acceptable.

Different studies have been published on the applications of NIRS in determining dry matter content in Hass avocados. These investigations span from 2002 to 2020. Other studies have demonstrated NIRS applications for the detection of bruises and the prediction of rot susceptibility in ‘Hass’ avocado fruit [34]. The progression of ripening has been monitored using NIRS to assess issues related to fruit transpiration [35], as well as postharvest classification strategies during avocado ripening, categorizing it by maturity stages based on firmness [36]. While these works are concerned with postharvest avocado quality, our focus here lies on research endeavors centered around the determination of dry matter content to ascertain the degree of maturity before or at the time of Hass avocado harvest.

The results of the PLS models obtained in this study for the calibration stage exhibited similarity in terms of the coefficient of determination found by various authors yet demonstrated enhanced precision performance with lower values of SEC and SECV compared to those reported in another research. This could potentially be attributed to the strategy of scanning different regions of the fruit to represent the inherent content and natural gradients present within it. The studies discussed subsequently focus on scanning the equatorial zone of the fruit. This observation was also noted in the validation phase, as is presented later.

Clark et al. were the first researchers to report the application of NIRS (300–1140 nm) on intact avocados using both reflectance and interactance modes. They conducted scans on the equatorial zone of the fruit. The researchers found SECV values of 2.6, which were higher than those reported in this study, with similar R^2^ values [10]. Blakey et al. predicted the moisture content in Hass avocados in South Africa (Tzaneen and Howick) over two harvest years (2007 and 2008). They scanned the fruits’ equatorial zone (400–2500 nm) and achieved an SEC value of 1.8 [20]. Wedding et al. observed higher SECV values of 1.41 in the calibration stage for Hass avocado samples from Australia (harvested from 2006 to 2008) sourced from different years (2006, 2007, 2008) on two commercial farms in Central Queensland. In their study, the fruits were scanned twice in the equatorial zone (830–2500 nm range) [19]. Olarewaju et al. conducted analyses in two orchards in KwaZulu-Natal, South Africa, between 2013 and 2014. Like the previous studies, they scanned the equatorial zone of the fruit (reflectance spectra were obtained at 2 nm intervals over the 700–2500 nm spectral range). The SEC values they found were lower than those reported by earlier authors (1.28) in the calibration stage but higher than those presented in this study [8]. Subedi and K.B. Walsh analyzed Hass avocado fruits sourced from four farms in different regions of Queensland, Australia, across the 2016, 2017, and 2018 seasons. Spectra and reference values were typically obtained from two sides of each fruit without specifying the scanning zone. These authors reported an RMSEC value of 1.72 [24].

**Figure 6:**
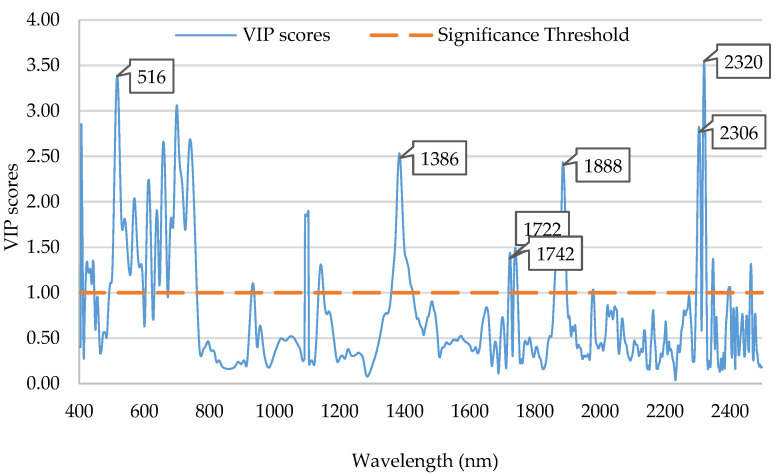
Variables important in projection (VIP) profiles along the whole NIR range of the selected PLS models developed to predict DM.

Figure 6 shows the VIP statistic of the PLS models developed to predict DM. The wavelengths with VIP values greater than 1 were considered significant and preferentially used. Therefore, the wavelengths assigned to a high (>1) VIP could potentially have a reasonable effect on the prediction and be selected for model estimation. The first absorption bands corresponded to the visible wavelength region (519–746 nm). The second absorption bands were between 930 and 1450 nm, with a peak at 1386 nm corresponding to water (O-H). The third overtone with further contribution from absorbance contained the bands for oil in the vicinity of 1722, 1742, and 1880 nm. Finally, the VIP significative bands were 2306 and 2320 nm, which were associated with OC, as discussed later.

The VIP > 1 results found were related mainly to the observed spectral peaks exhibiting similarity to those found in the Hass avocado [8,11,19,24,31]. Subedi and K.B. Walsh found that the peaks in the visible wavelength region (420–750 nm) were related to carotenoids (approximately 550 nm) and chlorophyll (approximately 680 nm) in Hass avocado skin [24]. Olarewaju et al. and Wedding et al. showed that the beta coefficients of PLS-R models for intact Hass avocado fruits were 970 and 1200 nm and closely associated with the H-O-H stretching modes of water [8,19]. The wavelength bands between 900 and 920 nm and at approximately 2200 and 2400 nm were associated with the C-H_2_ stretching and combinations related to oil.

#### 2.2.2. Tests of the Model Using Independent Validation Sets

The training and external validation of the models were carried out as follows: Learning using the Nápoles (2022a and 2022b) and Sotareño (2022a) datasets (N = 540) and using the orchard Recuerdo (2022b) dataset (N = 180) as an independent set for validation. Thus, the validation set corresponded to a new orchard.

**Figure 7:**
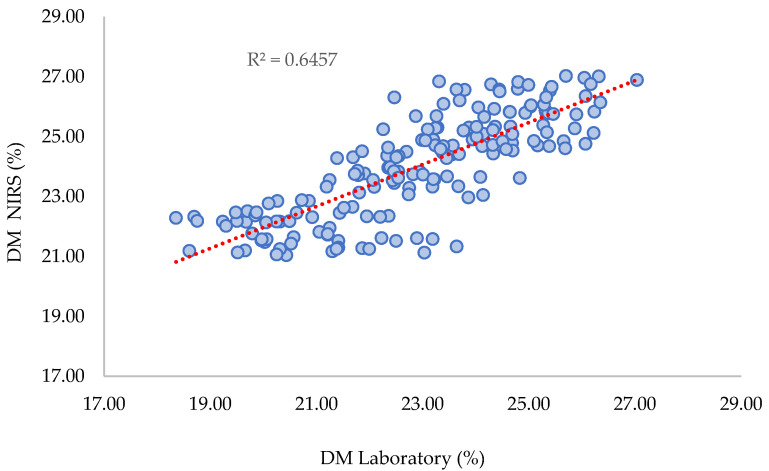
Scatter plot of DM laboratory values versus DM NIRS predicted values.

The summary statistics of the performances of the learning models are presented in Table 2. The SECV and R^2^ (see Figure 7) were similar to those observed for the general model (two harvest cycles: 2022a and 2022b), as well as the number of PLS LVs and outliers.

For the prediction of the Recuerdo orchard DM content, the standard error of prediction SEP was 1.62%, which was close to the SECV (1.15%), and there was only a correction of bias and slope applied. This is expected, as in this case, the Recuerdo orchard represents new and independent samples. This result demonstrates the high performance of the model and its accuracy. The bias and relatively low values for R^2^ (prediction) observed confirm that even if the accuracy of the models is efficient, the robustness of the model needs to be reinforced by adding samples from new harvests and new orchards. The decrease in the R^2^ value of NIRS models applied to the prediction of various avocado Hass farms or harvest seasons has also been reported by Wedding et al. [11,19,31]. The authors indicate that the dry matter content of avocados exhibits significant biological variability depending on the production region and harvest season.

Recent studies have indicated that fruit development time in days affects fruit quality upon reaching the destination market. Consequently, orchards that harvest Hass avocados within a short development period (162–176 days) tend to yield poor-quality fruits. Furthermore, a wide variation in dry matter content has been observed in commercial Hass avocado crops [37]. In Colombia, the DM content has been observed to range between 18 and 28% (120 days to 215 days of fruit development, respectively). The results showed that the field sampling methodology allowed monitoring of DM in this range by laboratory and NIRS methods. In this work, we found that the dry matter accumulation rate for the study farms was 0.071% per day (see Figure S4).

Based on these results, for monitoring dry matter during fruit development, it is fundamental in PLS models for Hass avocado quality studies performed by NIRS to have low values of SEC and SEP, in addition to having appropriate R^2^ values, since these statistics are used to evaluate the precision of the calibration model and the accuracy for future predictions [38]. According to the daily accumulation of DM, a SEP of 1% in the prediction corresponded to a deviation of approximately 14 days in the accuracy of the model’s prediction. This deviation, however, did not significantly impact the overall quality. On the other hand, models with high SEP values (>2%) resulting in a prediction error of more than 30 days would have a substantial impact on quality, particularly in terms of underestimating the dry matter content [37]. This scenario would result in harvesting fruits with insufficient maturity levels, failing to meet market expectations.

Previous research on the application of NIRS in estimating dry matter (DM) in Hass avocados with a single scan in the equatorial zone of the fruit yielded similar R^2^ values in calibration. Many of these studies were conducted by removing the fruit peel [10,19,31], while others were performed on the whole fruit [8,19,20] and found higher R^2^ values when scanning the fruits after removing the peel in the DM analysis, but that limited their application as a nondestructive measure of the maturity of Hass avocados. The results of this work were similar to the predictive performance of the model developed using multiple seasons in the calibration model of the avocado maturity parameter [11,19,31]. However, the results of this study for SEC (<1%) and SEP (<1.7) were lower than those reported by Olarewaju (3.13), Blake (3.78), and Clark (2.6) [8,10,20]. Considering the daily accumulation of dry matter, the results of this study showed a maximum estimation variation at 20 days, while for previously published studies, this difference could range from 36 to 53 days, which can impact harvest decisions and fruit selection. The low values of SEC, SECV, and SEP found in this study may be attributed to the strategy of conducting multiple samplings at different harvest times and performing multiple scans per fruit.

## 3. Materials and Methods

For this study, two experiments were conducted. The first experiment involved the establishment of a scanning methodology across distinct fruit zones (peduncle, equator, and base) using near-infrared spectroscopy (NIRS). The second experiment encompassed the implementation of this methodology throughout the fruit’s developmental stages across different orchards and harvest cycles.

### 3.1. NIRS Scans Per Fruit Zone for Assessing the Dry Matter Gradient in Whole Fruit

#### 3.1.1. Avocado Sampling

For this experiment, ten Hass avocados (*Persea americana* Mill cv. Hass) fruits were collected randomly in a packing house line (Pacific Fruits, Palmira Colombia) following the procedures published to the sampling fruit at orchards and packing houses for the reference’s laboratory method [39,40]. Fruit samples were marked with a number from 1 to 10. NIR spectra analysis was collected in the CIAT facilities (International Center for Agriculture Tropical Palmira Colombia) in the Nutritional Quality Laboratory (NQL), and DM quantification was made, using the oven method, in the Palmira Research Center of Agrosavia (Colombia).

#### 3.1.2. Spectroscopic Measurement and Data Acquisition

**Figure 8:**
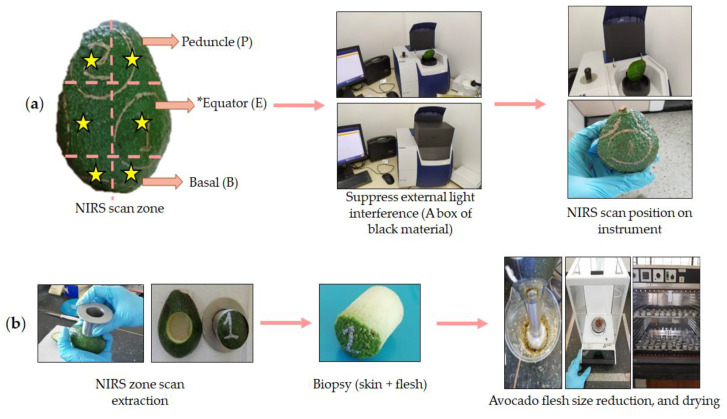
Scheme of NIRS spectra acquisition on the surface of a whole Hass avocado fruit (**a**); DM quantification using oven drying (**b**). * “Equator” is used to refer to the centerline of the main body of the fruit [24].

The spectra were obtained using a near-infrared FOSS DS 2500 spectrometer device (FOSS, DK-3400, Hilleroed, Denmark). Each fruit was analyzed in three zones: the peduncle, equator, and base (Figure 8a). For each zone, in a different fruit side, three measurements were performed on each intact Hass avocado (peduncle, equator, and base). The fruit was placed on a round capsule sample holder with an external diameter of 5 cm and a quartz window with an internal diameter of 3.8 cm for spectral reading in reflectance mode. A box of black material was adapted in the NIRS scan zone of the equipment to avoid interference from external light (Figure 8a). All reflectance NIR spectra were obtained at 2 nm intervals from 400 to 2500 nm in the wavelength range. Each spectrum consisted of 32 scans that were automatically averaged and recorded as log 1/reflectance (log 1/R). Spectral data were extracted with the WinISI Version 4.6.8 program (FOSS, DK-3400, Hilleroed, Denmark).

### 3.2. Application of the Method of Different NIRS Scans by Zone in Maturity Monitoring of Hass Avocado

#### 3.2.1. Avocado Fruit Sampling

For the application of the previously presented methodology, samples were collected from commercial orchards located in the Cauca Department (Colombia), specifically in the municipalities of Morales (orchard Recuerdo), El Tambo (orchard Nápoles), and Sotará (orchard Sotareño). Samples were collected in two harvest cycles in 2022, referred to as 2022a and 2022b. The 2022a harvest took place between 30 March and 7 June in the Nápoles and Sotareño orchards. The 2022b harvest occurred between 21 September and 29 November in the Nápoles and Recuerdo orchards. During these periods, the fruit was collected at six different stages of development to monitor dry matter content (commercial harvest in Colombia typically includes fruit with a dry matter range of 18–30% [6]). Ten fruits were collected for each stage of fruit development, resulting in a total of 60 fruits per orchard and 120 fruits per harvest cycle. Therefore, a total of 240 fruits were analyzed across both harvest cycles.

After sampling, the fruits were packed in perforated kraft paper bags and transported to the laboratory on the same day in styrofoam boxes with separate refrigerant gel packs to prevent cold damage to the fruit. In the laboratory, prior to analysis, the fruits were stored under refrigeration at 5 °C.

#### 3.2.2. Spectroscopic Measurement and Data Acquisition

As discussed in the results and discussion section, it was found that one spectrum per fruit zone could effectively represent the dry matter (DM) gradient in the entire fruit. Consequently, three zones (peduncle, equator, and base) were scanned for each fruit. Data acquisition followed the previously presented methodology. Overall, a total of 720 NIRS scans (240 fruits × 3 scans) and corresponding DM laboratory analyses were conducted across the two harvest cycles.

### 3.3. Dry Matter Analysis with the Reference Method

The same area of the fruit scanned was labeled with a metallic marker (Artline 990XF gold; Nagoya City, Japan), and then, a 3 cm diameter core that was perpendicular to the surface of the fruit (skin + flesh) was extracted using a steel corer (9 cores were taken per fruit). The skin was included in the analysis because it was part of the spectral acquisition. Moreover, the exocarp contained sample-dependent oil and moisture contents that encountered radiation from the spectrometer during scanning [21]. Next, the fresh mass of each cut sample was processed with an immersion blender (KHB2561OB 5-Speed, KitchenAid; Mississauga, ON, Canada) to obtain a homogeneous extract. Samples were dehydrated using a hot air oven (UL 50, Memmert, Schwabach, Germany) at a temperature of 75 °C for 48 h until reaching a constant weight [21]. The dry matter (DM) content was calculated based on Equation (1):

DM (%) = (W1/W2) × 100 (1)

where W1 refers to the weight of the oven-dried sample (g), and W2 is the weight of the wet avocado sample.

### 3.4. Data Analysis

#### 3.4.1. DM Descriptive Statistics and ANOVA

For the first experiment of the work to evaluate the effect of zone effect on DM fruit content, descriptive statistics and DM distribution (histogram) were calculated. For data analysis, a two-way ANOVA (fruit and scanning zone) was employed. Subsequently, a Newman–Keuls post hoc test (SNK: Student–Newman–Keuls) was applied for means comparison, with a 95% confidence interval. All statistics were performed using XLSTAT software (Addinsoft (2023)). XLSTAT statistical and data analysis solution (Paris, France, https://www.xlstat.com/fr, accessed on 28 August 2023).

#### 3.4.2. Chemometric Data Analysis

(a) Number of Scans per Fruit zone.

The principal component analysis (PCA mean-centered) was performed with the raw data of the whole spectra. Cross-validation was performed by applying a random model with 20 segments using a singular value decomposition (SVD) algorithm.

Spectrum quality was evaluated using the root mean square (RMS) statistic [41,42]. The RMS statistic included in the WinISI software was used to calculate the agreement between the spectra within the Hass avocado fruit zone and between them.

(b) Application of different NIRS scans by fruit in Maturity Monitoring of Hass Avocados.

For harvest 2022a and 2022b, the calibration models and chemometrics were realized using WinISI IV software (FOSS DK-3400 Hilleroed, Denmark). Different pretreatments were tested. Then, the first derivative was calculated on four data points and smoothed using Savitzky and Golay polynomial smoothing on four data points on the whole dataset, including visible and near-infrared wavelengths [43]. The calibration equations for dry matter content were developed using the modified partial least-squares regression (mPLS) algorithm of WinISI software [44].

The variable importance in the projection (VIP) measure was computed as the summation of the squared PLS X-score coefficients for the selected components; the weights were derived from the extent of variance in the dependent variable (y) that was accounted for by each of the said components. Thus, the VIP statistics effectively confirmed the impact of a given variable on the PLS model [45]. The variable importance in the projection values (VIP > 1) in PLS projection was calculated to estimate the contribution of each wavelength of the NIR spectra to the DM prediction. The VIP-based variable selection methods were implemented with custom routines in MATLAB (MATLAB, the MathWorks, Natick, MA, USA).

Cross-validation using 4 groups was applied during calibration development to select the optimum number of latent variables. The statistics used to describe the models were the standard deviation (SD), coefficient of determination (R^2^), standard error of calibration (SEC), and standard error of cross-validation (SECV). Student’s t-test was used to identify t-outlier samples during calibration development. Outlier detection was based on the standardized residuals (=error/SECV) with a cutoff of 2.5. Two passes of outlier elimination were used [46]. The best performances for the model correspond to the first derivative (Norris derivative) calculated using an applied to the spectra corrected using the detrend function. The first-order Norris derivative was calculated using a gap equal to 4 (data points), and after derivation, smoothing (polynomial of second order) was applied to the curve using a gap of 4. The whole spectrum, 400 nm to 2500 nm, was used, which corresponded to 1050 data points.

## 4. Conclusions

In the first part of the work, the laboratory determination (destructive method) and analysis of NIRS confirmed the heterogeneous repartition of dry matter within the fruit. There was a significant effect on the fruit zone (equator, base, and peduncle). The peduncle stands out as the region with the highest dry matter content. The raw spectra and PCA analysis corroborate this trend. The RMSi showed the existing spectral variability between the fruit zones and demonstrated that spectral variability per fruit area could be captured with a single spectrum. This methodology allowed promising values to be found during calibration and validation with independent samples, especially in relation to the precision and accuracy of DM prediction due to its low SEC, SECV, and SEP values. In future investigations, it is crucial to consider the dry matter gradient inherent in the fruit, necessitating the inclusion of various zones in NIRS scans to enhance precision. Moreover, future studies must incorporate fruit physiological variability during fruit development and more orchards.

## Figures and Tables

**Table 1:** Results of ANOVA sum of squares type III of de DM.

Factors	Degrees of Freedom	Sum of Squares	Mean Squares	Fisher’s Exact Test	* Pr > F
Fruit	9	35	4	282	0
Zone	2	2	1	56	0

* Pr > F: probability corresponding to the Fisher’s (F) test.

**Table 2:** Performance of the PLS models using independent validation sets.

	Harvest	Calibration Nápoles, Sotareño 2022a and Nápoles 2022b_Validation Recuerdo 2022b
Statistics		
N calibration		527
N prediction		180
Range DM Laboratory		18.73–27.42
SD		2.005
SEC		0.995
R^2^		0.754
Range NIR Prediction		18.35–27.04
SECV		1.151
#LV		12
# Outliers		13
SEP		1.62
Bias		1.10
SEPc		1.19
Slope		0.70
R^2^		0.70

N: number of scans; SD: standard deviation; SEC: standard error of calibration; R^2^: coefficient of determination; SECV: standard error of cross-validation; #LV: number of latent variables in the PLS model; SEP: Standard error of prediction; SEPc: Standard error of calibration prediction.

## Data Availability

The data that support the findings of this study are available from the corresponding author upon reasonable request.

## Supplementary Materials

The following supporting information can be downloaded at: https://www.mdpi.com/article/10.3390/plants12173135/s1, Figure S1: Variability in the dry matter percentage in the Hass avocado samples. Figure S2: RMSi (root mean squared for each spectra) values in µabs for all 3 spectra per zone per fruit. Figure S3: RMSi (root mean squared for each spectra) mean and standard error values between Hass avocado fruit zones. Figure S4: Relationship between fruit development time and average dry matter content over time for the orchards evaluated in the two harvest cycles.

## List of Abbreviations

**Abbreviations**	**Meaning**
NIRS	Near-infrared spectroscopy
DM	Dry matter
OC	Oil content
PCA	Principal component analysis
PLS	Partial least-squares
SNK	Student Newman–Keuls
SVD	Singular value decomposition
GAP	Gap difference method
RMS	Root mean squared
RMSi	Root mean squared for each NIRS spectra
RMSEC	Root mean squared error of calibration
RMSECV	Root mean square error of cross-validation
RMSEP	Root mean squared error of prediction
SEC	Standard error of calibration
SECV	Standard error of cross-validation
SEP	Standard error of prediction
LV	Latent variables in the PLS model
