# Supplementary material for: Quantification of Dry Matter Content in Hass Avocado by Near-Infrared Spectroscopy (NIRS) Scanning Different Fruit Zones
Source: Plants (Basel). 2023 Aug 31;12(17):3135. doi: 10.3390/plants12173135 (PMC10490472; doi:10.3390/plants12173135)
Supplement: Supplementary file 1 [file plants-12-03135-s001.zip › plants-2548679-supplementary.pdf]

Supplementary material

Quantification of Dry Matter Content in Hass Avocado by Near-Infrared Spectroscopy (NIRS) scanning different Fruit Zones

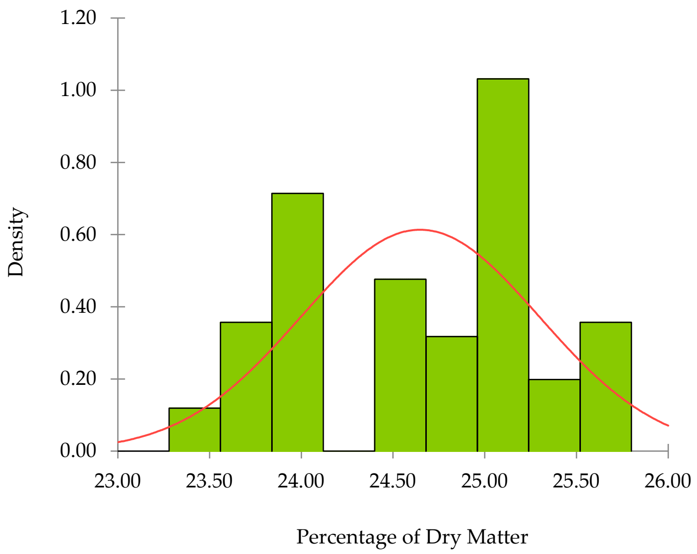

Figure S1. Variability in the dry matter percentage in the Hass avocado samples. The red line represents the normal distribution curve density with a mean of 24.64% DM and a standard deviation of 0.65% DM.

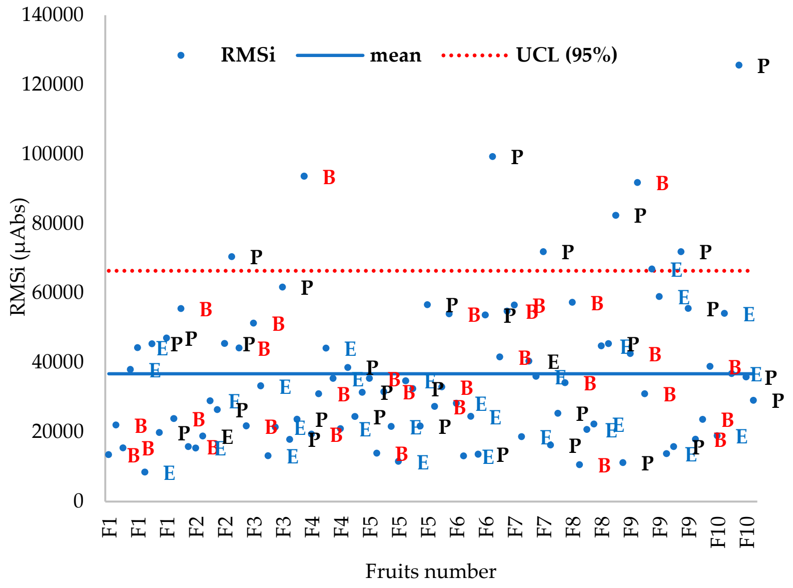

Figure S2. RMSi (Root Mean Squared for each spectra) values in µabs for all 3 spectra per zone per fruit. Fruit zone: B: base, E: equator, P: peduncle. F: Fruit number.

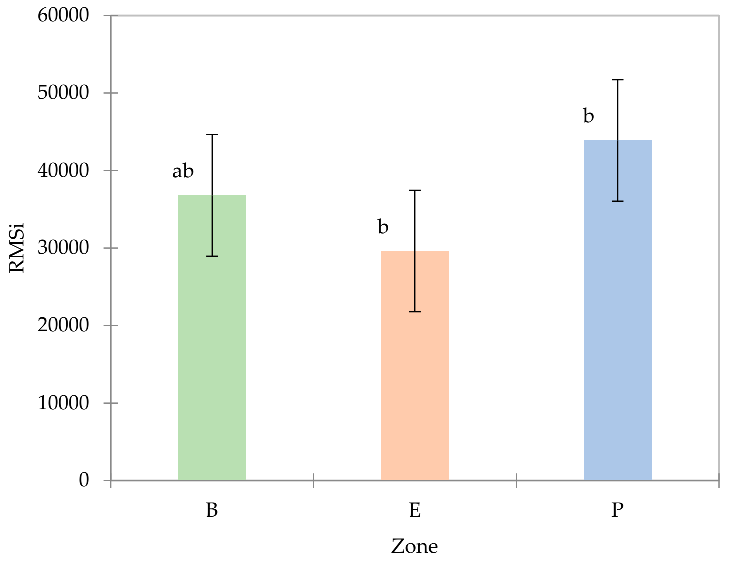

Figure S3. RMSi (Root Mean Squared for each spectra) mean and standard error values between Hass avocado fruit zones. Different letters represent significant differences among the fruit zone (Test Chi-square ($p < 0.05$)). Fruit zone: B: base, E: equator, P: peduncle.

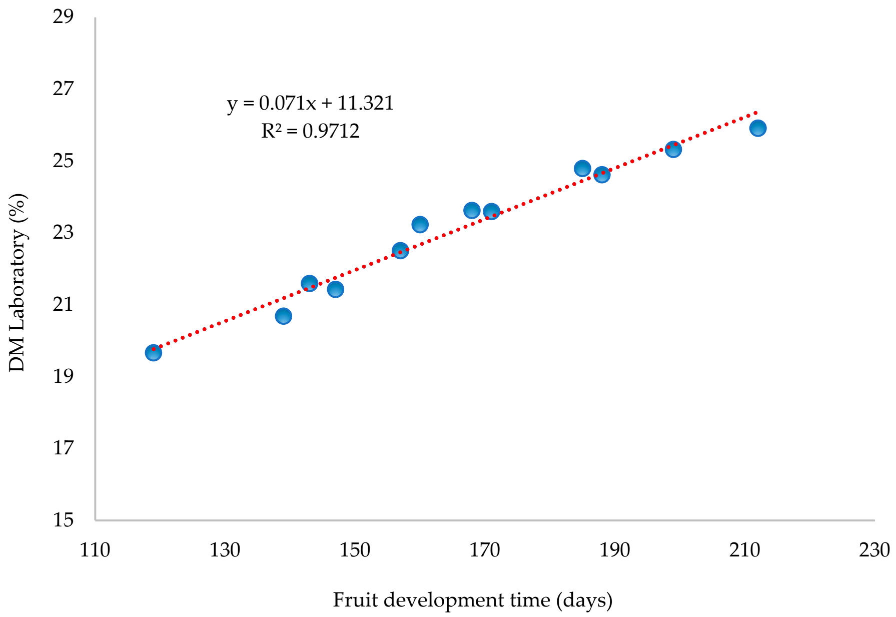

Figure S4. Relationship between fruit development time and average dry matter content over time for the orchards evaluated in the two harvest cycles.
